# Effect of Paper-Bagging on Apple Skin Patterning Associated with *MdMYB10* Promoter Methylation

**DOI:** 10.3390/ijms23063319

**Published:** 2022-03-19

**Authors:** Hye Jeong Cho, A Reum Han, Cheol Choi

**Affiliations:** College of Agriculture and Life Sciences, Kyungpook National University, 80, Daehak-ro, Buk-gu, Daegu 41566, Korea; hyejeong725@naver.com (H.J.C.); har0903@naver.com (A.R.H.)

**Keywords:** anthocyanin, apple, bag treatment, DNA methylation, MYB transcription factor, skin patterning

## Abstract

Paper-bagging is an efficient method to maximize apple skin color, but a relationship between this technique and fruit skin patterning has not been demonstrated. Here, the ‘Fuji’ fruit with red-striped skin changed to red-blushed skin under re-exposure to light after bag treatment. Higher expression of *MdMYB10*, a transcription factor that regulates anthocyanin biosynthesis in apples, correlated with increased anthocyanin concentration in bag removal fruit. At the mature stage, a comparison of methylation status in the *MdMYB10* promoter revealed that the methylation level in the region from −2585 to −2117 bp was reduced in bag removal fruit, especially for CHG context. It can be regulated by the downregulated expression of DNA methyltransferases such as *MdMET*, *MdCMT*, and *MdDRM*. Our results suggest that the bag removal treatment in this cultivar causes a change in skin patterning from striped to blushed pigmentation by inducing DNA demethylation of *MdMYB10*.

## 1. Introduction

‘Fuji’ apples originated as a cross between the ‘Ralls Janet’ and ‘Red Delicious’ varieties [1]. The popularity of ‘Fuji’ apple is increasing because of the fruit’s sweet flavor, firmness, and visual appearance [2]. ‘Fuji’ apples are categorized as having a striped skin pattern because the harvested fruit typically has bi-colored yellow and red stripes [3]. In contrast, most other apple cultivars have a defined fruit pigment. The red highlights of ‘Fuji’ apples are not uniform when cultivated under different conditions, such as high temperature or decreased light [4]. The molecular basis for these skin patterning phenomena has not been elucidated.

Anthocyanin is a phenolic compound synthesized via the flavonoid pathway and is responsible for red-colored fruit such as apple, pear, grape, peach, and strawberry [5]. Its synthesis is regulated by genetic and environmental factors [6]. For example, the expression of multiple regulatory and structural genes involved in anthocyanin synthesis in this fruit is enhanced by sunlight [7]. Light intensity and temperature are the two most critical environmental factors affecting anthocyanin accumulation in the apple peel [8]. Previous work has shown that anthocyanin preferentially accumulates in apple skin exposed to UV-B visible light rather than shade [9].

Various agronomic approaches have been developed to modulate light exposure, such as fruit bagging, leaf removal, and reflective film. Fruit bagging treatment is primarily a process of controlling light exposure, although air composition, temperature, and humidity conditions around the fruit may also change, resulting in various physiological responses [10]. Bagging has been used to improve outer and inner fruit traits, including skin coloration, sweetness, and acidity. Because the accumulation of flavonoid and anthocyanin improves fruit color development and is more sensitive to light than other characteristics, the effects of bagging have been evaluated for these characteristics. Bagging of apples during fruit development with bag removal at fruit maturation increases the amount of anthocyanin accumulation and the activity of related enzymes. Therefore, fruit bagging is widely used to promote anthocyanin synthesis and improve fruit coloration in apples [11]. It is believed that bagging increases the light sensitivity of fruit, and re-exposure to light after bag removal stimulates anthocyanin synthesis.

Results of paper-bagging vary from species to species and even between cultivars of the same species. In ‘Red Fuji’ and ‘Fuji Suprema’ cultivars, bagging causes improved calcium absorption, a lower incidence of bitter pit, and a higher incidence of russeting [12,13]. In ‘Fuji Raku Raku’, bagging increases antioxidant capacity [14]. However, the focus of most studies has been traits like calcium and phenolic compound accumulations and not changes in pigmentation. In other cultivars, regardless of whether the fruits are green- or red-skinned, bag removal causes a rapid change to red [15,16]. The mechanism of how light-dependent pigmentation is regulated is not understood, but it has been proposed that light stimulation affects DNA methylation and enhances fruit redness.

DNA methylation refers to methyl groups at the 5′ position of cytosine (5 mC). Depending on neighboring nucleotides, DNA methylation is classified as symmetric (CG or CHG) or asymmetric (CHH). DNA methylation is catalyzed by DNA methyltransferases, such as methyltransferase (*MET*), chromomethyltransferase (*CMT*), and domain rearranged methyltransferase (*DRM*) [17]. The activities of these DNA methylating enzymes suggest that reversibility of DNA methylation is critical for regulating specific genes during development. *CMT* is a plant-specific DNA methylation enzyme, but all three DNA methyltransferases (*MET*, *CMT*, and *DRM*) have been proposed to regulate apple fruit coloration by regulating DNA methylation [18].

The methylation status of *MdMYB1*/*10* genes is responsible for color and pattern variation in apple cultivars such as ‘Honeycrisp’ and ‘Gala’ strains [19,20]. In the non-red cultivars ‘Mutsu’ and ‘Granny Smith’, red pigmentation was induced by paper-bagging and epigenetic regulation of *MdMYB1* by 5-aza-2′-deoxycytidine [21,22]. The transcription levels of DNA methyltransferases were examined for the first time in ‘Granny Smith’ apple by RNA-sequencing [18]. The yellow-striped areas in ‘Fuji’ strains are thought to have a more highly methylated *MdMYB1*/*10* promoter than a red-striped area [23,24,25].

Although the effect of paper-bagging on skin appearance in apples has been studied, there are few reports about paper-bagging’s impact on skin patterning to date. The focus of this study was to elucidate the mechanism of epigenetic regulation in striped-skinned ‘Fuji’ apples following paper-bagging.

## 2. Results

### 2.1. Anthocyanin Analysis

**Figure 1:**
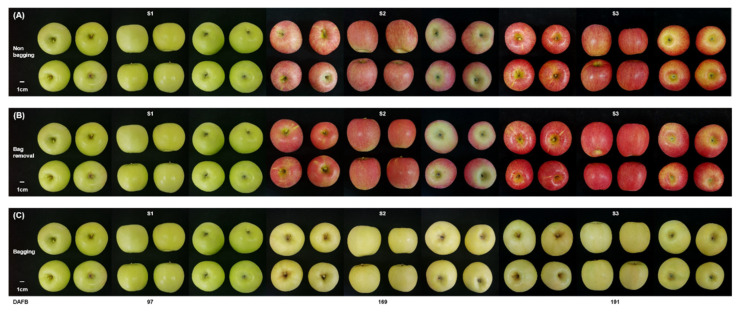
Skin color and pattern changes in ‘Fuji’ apples subjected to (**A**) no bagging (control; labeled as “non-bagging”), (**B**) bagging followed by bag removal (labeled as “bag removal”), and (**C**) bagging with no bag removal (labeled as “bagging”). Apples were harvested at three time points: before paper-bagging (S1, DAFB 97) and then 1 and 4 weeks after bag removal (S2, DAFB 169 and S3, DAFB 191). At maturity (S3), ‘Fuji’ apples display a striped-red color (**A**), a blushed-red color (**B**), and a blushed-yellow color (**C**), depending on bagging treatment. Scale bars equal 1 cm. DAFB, days after full bloom.

The role of bag treatment in the red-striped ‘Fuji’ fruit was examined. Three groups were analyzed in this study: fruit labeled as “non-bagging” were not subjected to paper-bagging; fruit labeled “bag removal” were paper-bagged, and then the bag was removed; fruit labeled as “bagging” were paper-bagged, but the bag was not removed. In the bag removal group, the fruit color changed to red-blushed skin, whereas bagging fruit stayed light yellow (Figure 1).

**Figure 2:**
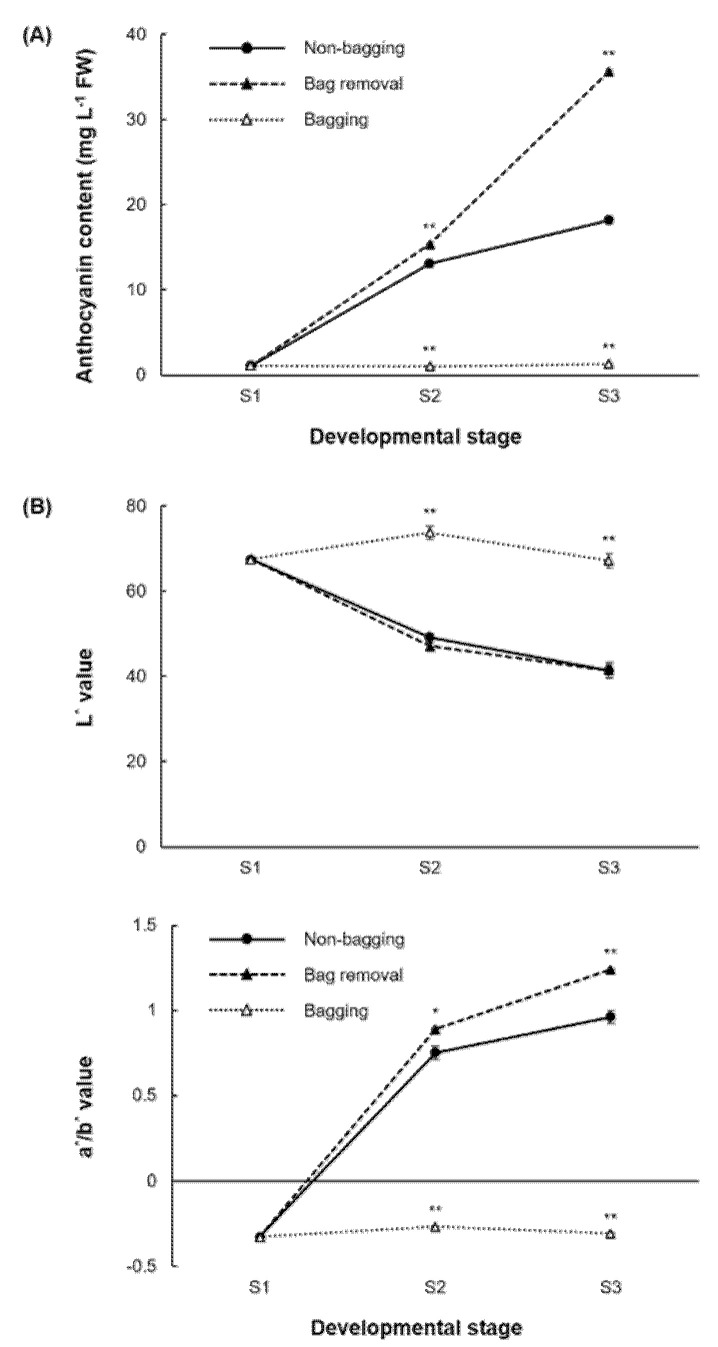
Measurements of (**A**) anthocyanin accumulation and (**B**) color parameters (L* and a*/b*) in the skin of ‘Fuji’ apples cultivated under different bagging treatments: no bagging (control; labeled as “non-bagging”), bagging followed by bag removal (labeled as “bag removal”), and bagging with no bag removal (labeled as “bagging”). Apples were harvested at three time points: before paper-bagging (S1, DAFB 97) and then 1 and 4 weeks after bag removal (S2, DAFB 169 and S3, DAFB 191). Bars represent the standard error of triplicate samples. * and ** indicate significance levels of *p* < 0.05 and *p* < 0.01, respectively. DAFB, days after full bloom.

Each pigmentation pattern was associated with a different level of anthocyanin content (Figure 2A). The total anthocyanin in fruit skin was calculated using cyanidin-3-galactoside, a predominant compound in apples. In non-bagging apples, the level of anthocyanin increased steadily from S1 to S3, whereas the anthocyanin accumulation behavior is different in bag-treated apples. Relative to non-bagging fruit, the anthocyanin content in bag removal fruit is significantly elevated at all developmental stages (S1–S3), while it is significantly reduced in bagging fruit. The most significant differences appear at S3.

Because paper-bagging induces a change in skin color and pattern, L*, a*, and b* values were calculated over the entire surface of fruit skins using the Tomato Analyzer software (Figure 2B). L* and a*/b* values represent lightness and redness, respectively. A pattern similar to previous anthocyanin content was seen in L* and a*/b* data. Specifically, the L* value was significantly higher in bagging fruit, and the a*/b* value was higher in bag removal fruit.

### 2.2. Expression of the Anthocyanin-Related Genes

**Figure 3:**
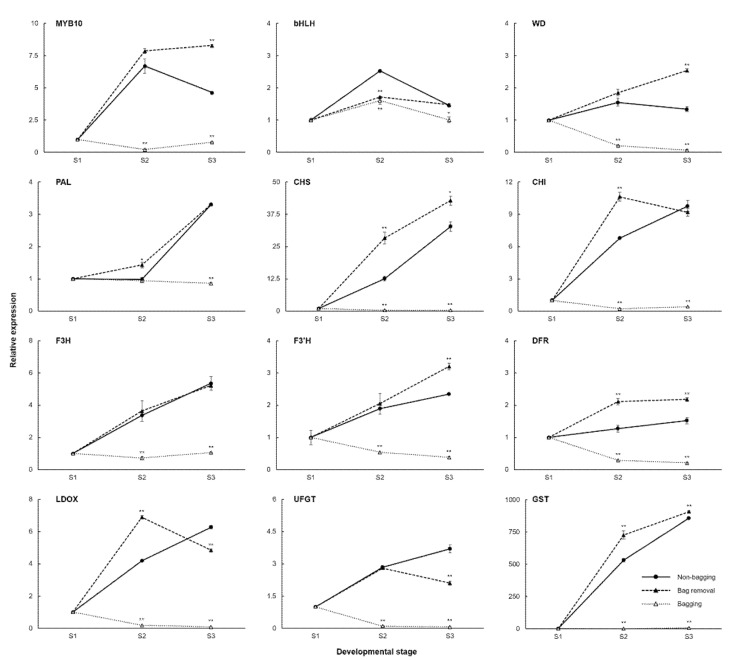
Quantitative real-time PCR analysis of anthocyanin-related genes in ‘Fuji’ apples cultivated under different bagging treatments: no bagging (control; labeled as “non-bagging”), bagging followed by bag removal (labeled as “bag removal”), and bagging with no bag removal (labeled as “bagging”). Apples were harvested at three time points: before paper-bagging (S1, DAFB 97) and then 1 and 4 weeks after bag removal (S2, DAFB 169 and S3, DAFB 191). The *x*-axis and *y*-axis indicate the time of harvesting and relative expression, respectively. For each gene, the expression level at S1 was set as 1. Bars represent the standard error of triplicate samples. * and ** indicate significance levels of *p* < 0.05 and *p* < 0.01, respectively. DAFB, days after full bloom.

The anthocyanin pathway in apple consists of regulatory (*MdMYB10*, *MdbHLH*, and *MdWD*), structural (*MdPAL*, *MdCHS*, *MdCHI*, *MdF3H*, *MdF3′H*, *MdDFR*, *MdLDOX*, and *MdUFGT*), and transport (*MdGST*) genes (Figure S1). We measured and analyzed the expression levels of these genes during fruit color development by quantitative real-time PCR. The findings for bag removal and bagging fruit were compared to those for non-bagging fruit (Figure 3). The expression levels of anthocyanin-related genes in bag removal and non-bagging fruit show similar trends. Most genes were upregulated from S1 to S3 and peaked at S3, but most genes in bagging fruit display opposite expression patterns because bagging fruit do not accumulate anthocyanin due to a lack of light exposure. In bag removal fruit, regulatory genes *MdMYB10*, *MdWD*, and *MdGST* and all structural genes were significantly upregulated by re-exposure to light. The most significant difference appears in *MdMYB10* expression. Although *MdPAL*, *MdCHI*, *MdF3H*, *MdLDOX*, and *MdUFGT* expression levels are lower than non-bagging fruit, expression levels of *MdPAL*, *MdCHI*, and *MdF3H* are similar at S3. In bagging fruit, most regulatory and structural genes were hardly expressed.

### 2.3. Effects of Bagging Treatment on the Methylation Level of MdMYB10 Promoter in ‘Fuji’ Apples

**Figure 4:**
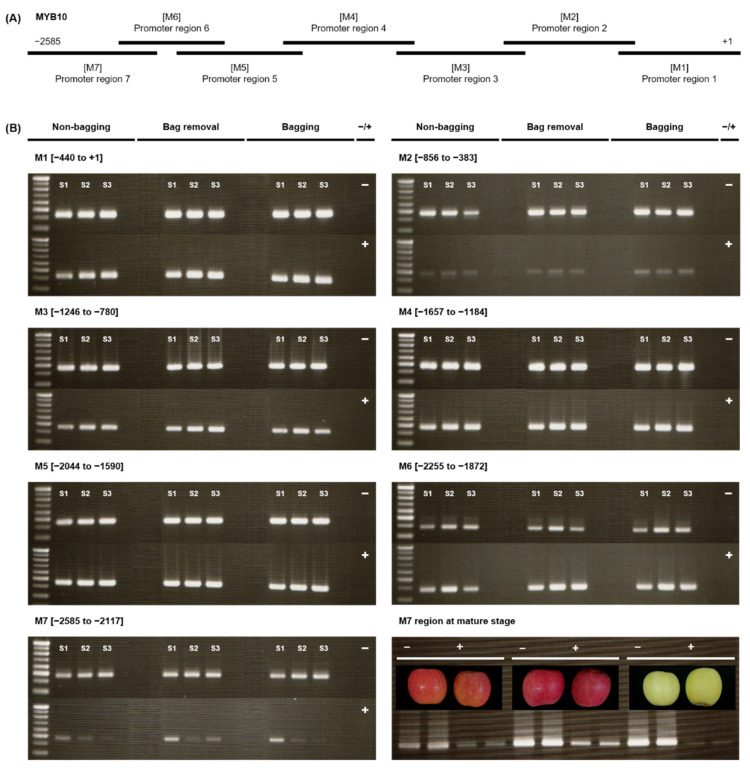
Changes in DNA methylation in the promoter of *MdMYB10* during development, effects of bagging treatment, and correlation with ‘Fuji’ apple skin patterning. (**A**) Schematic diagram of 2500 bp upstream of the ATG translational start site of *MdMYB10* and its division into seven overlapping fragments: M1-M7. (**B**) McrBC-PCR analysis where 500 ng of genomic DNA was digested with McrBC in the presence of GTP (+) or the absence of GTP (−). The absence of GTP served as the negative control. ‘Fuji’ apples were cultivated under different bagging treatments: no bagging (control; labeled as “non-bagging”), bagging followed by bag removal (labeled as “bag removal”), and bagging with no bag removal (labeled as “bagging”). Apples were harvested at three time points: before paper-bagging (S1, DAFB 97) and then 1 and 4 weeks after bag removal (S2, DAFB 169 and S3, DAFB 191). The panel labeled “M7 region at mature stage” shows the apple skin patterning: non-bagging, striped red; bag removal, blushed red; bagging, blushed yellow; +1, ATG translational start site. The last panel shows the technical replication of each treatment in the M7 region at the mature stage (S3). DAFB, days after full bloom.

The expression level of *MdMYB10* transcription factor can be regulated by DNA methylation in the promoter region. To examine the link between DNA methylation and apple skin patterning, the methylation level in the promoter region of *MdMYB10* was determined for non-bagging, bag removal, and bagging fruit. The entire promoter region (from −2585 to +1) was divided into seven fragments (M1-M7) (Figure 4A), and each segment was then digested at methylated cytosine residues by McrBC restriction enzyme. Methylation levels were evaluated during fruit color development (S1-S3) by measuring the brightness of bands between control samples (−) and McrBC-treated samples (+) (Figure 4B). M1 (−440 to +1), M3 (−1246 to −780), M4 (−1657 to −1184), M5 (−2044 to −1590), and M6 (−2255 to −1872) regions consistently exhibited low methylation at all stages under all bag treatments. The M2 region (−856 to −383) exhibited high methylation activity, resulting in almost complete digestion of the amplified DNA at all stages (S1–S3), regardless of bag treatment. However, the methylation level of the M7 region (−2585 to −2117) differs among the three bag treatments. DNA methylation levels gradually increase from S1 to S2 in a trend like the M2 region, but a recovery of methylation is detected at maturity (S3) in certain bag treatment. The brightness of the band from bag removal fruit is more intense than the non-bagging sample band. Moreover, there is no band in bagging fruit. These visible variations in methylation are also seen in the three replicates of mature stage fruit (M7 region at mature stage, Figure 4B). Therefore, the M7 promoter region was selected for further analysis.

**Figure 5:**
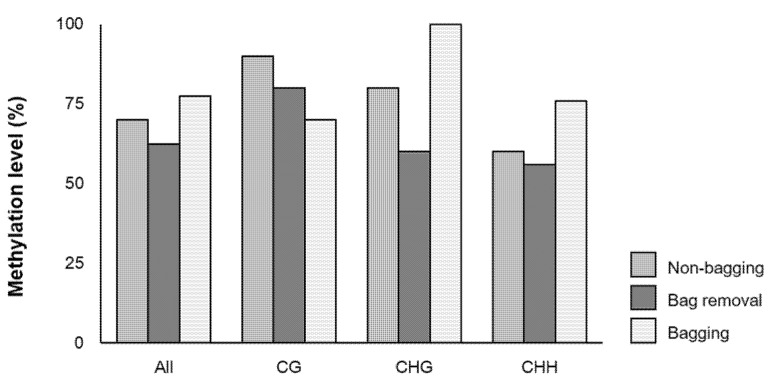
Cytosine methylation levels in the M7 region of *MdMYB10* promoter in mature ‘Fuji’ apples cultivated under different bag treatments: no bagging (control; labeled as “non-bagging”), bagging followed by bag removal (labeled as “bag removal”), and bagging with no bag removal (labeled as “bagging”). The *x*-axis and *y*-axis indicate different cytosine contexts (CG, CHG, and CHH) and methylation level, respectively. For each bag treatment, ten clones were detected by bisulfite sequencing and analyzed using the CyMATE software. “All” refers to total methylated cytosine while H indicates A, C, or T.

At least ten clones for each bag treatment were used to analyze the cytosine methylation level of the M7 region at the mature stage. There is no sequence difference in the three bag treatment groups except for a few sequencing errors and then a total methylated cytosine and three cytosine contexts (i.e., CG, CHG, and CHH) were detected by bisulfite sequencing (Figure 5). The results show that the total methylation level was lower in bag removal fruit (62.5%) than non-bagging fruit (70.0%) followed by bagging fruit (77.5%). This methylation pattern is similar to CHG and CHH patterns except for the CG pattern, where the methylation level was higher in non-bagging fruit than bag removal fruit followed by bagging fruit. In particular, the CHG pattern exhibited the greatest difference in the three bag treatment groups with an average of 60.0% in bag removal fruit, followed by non-bagging fruit (80.0%) and bagging fruit (100.0%).

### 2.4. Methyltransferase Expression

**Figure 6:**
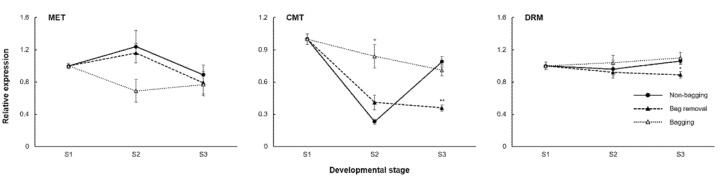
Quantitative real-time PCR analysis of methylation-related genes in ‘Fuji’ apples cultivated under different bagging treatments: no bagging (control; labeled as “non-bagging”), bagging followed by bag removal (labeled as “bag removal”), and bagging with no bag removal (labeled as “bagging”). Apples were harvested at three time points: before paper-bagging (S1, DAFB 97) and then 1 and 4 weeks after bag removal (S2, DAFB 169 and S3, DAFB 191). The *x*-axis and *y*-axis indicate the time of harvesting and relative expression, respectively. For each gene, the expression level at S1 was set as 1. Bars represent the standard error of triplicate samples. * and ** indicate significance levels of *p* < 0.05 and *p* < 0.01, respectively. DAFB, days after full bloom.

DNA methyltransferases mediate the partial DNA methylation observed in the *MdMYB10* promoter. Quantitative real-time PCR was conducted to measure *MdMET*, *MdCMT*, and *MdDRM* expression levels in the three bag treatment groups throughout the coloring period. The findings for bag removal and bagging fruit were compared to those for non-bagging fruit (Figure 6). *MdMET* expression level in bag removal and non-bagging fruit displays a similar trend from S1 to S3. *MdMET* is upregulated from S1 to S2 and downregulated in S3. *MdMET* expression in bag removal fruit is lower than in non-bagging fruit, although the difference did not reach statistical significance. *MdCMT* and *MdDRM* expression levels in bag removal and non-bagging fruit display similar trends from S1 to S2. *MdCMT* and *MdDRM* genes are downregulated from S1 to S2. At S3, *MdCMT* and *MdDRM* expression is significantly downregulated in bag removal fruit and upregulated in non-bagging fruit. No consistent pattern between bagging and non-bagging fruit is observed, and differences do not reach statistical significance. Although *MdMET* expression is lower in bagging fruit than non-bagging fruit, it peaks at S3 when the expression in bag removal fruit is downregulated. Overall, expression levels of *MdCMT* and *MdDRM* in the bagging group are higher than in non-bagging and bag removal groups.

### 2.5. Visualization of Whole Gene Expression Using Heat Map Software

**Figure 7:**
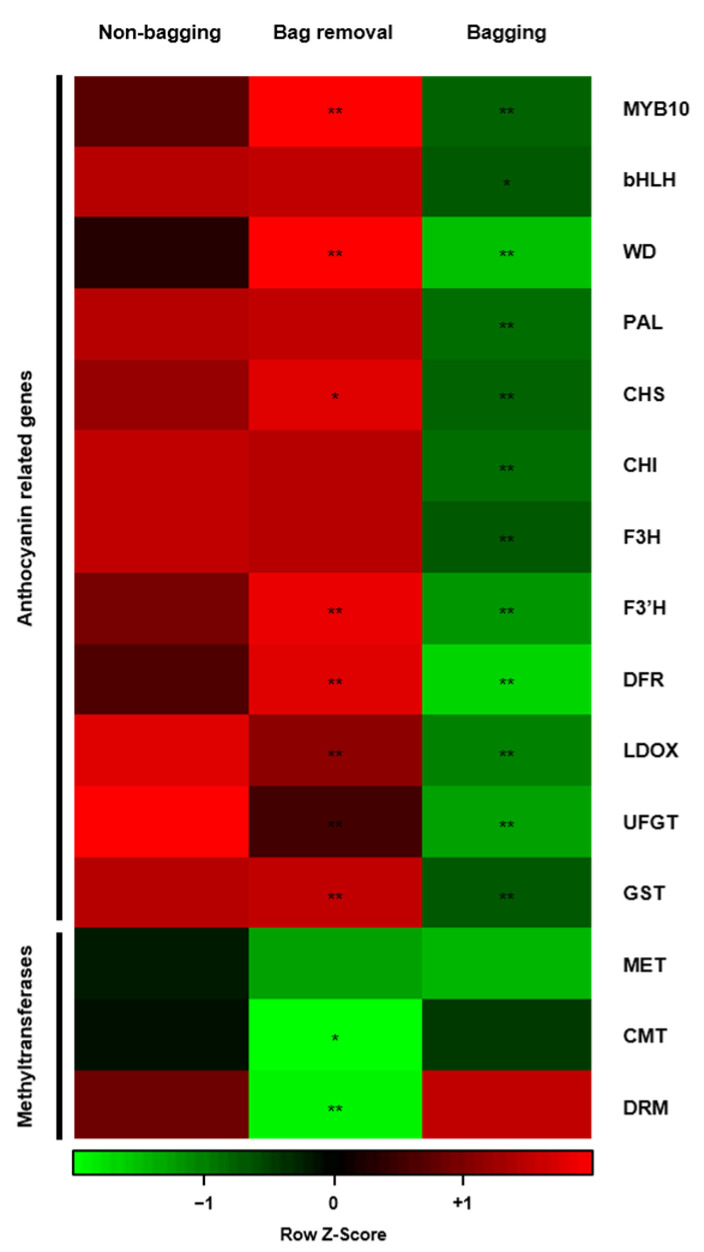
Heat map analysis for expression levels of anthocyanin- and methylation-related genes in mature ‘Fuji’ apples cultivated under different bag treatments: no bagging (control; labeled as “non-bagging”), bagging followed by bag removal (labeled as “bag removal”), and bagging with no bag removal (labeled as “bagging”). The color scale at the bottom shows a log_2_-transformed gene expression level from −1 (green), through 0 (black), to +1 (red) that represents low, intermediate, and high expression levels. * and ** indicate significance levels of *p* < 0.05 and *p* < 0.01, respectively.

Expression patterns of anthocyanin biosynthesis and methyltransferase genes were visualized in response to three bag treatments using heat map analysis to investigate the relationship between light-induced pigmentation patterns and whole gene activity at mature stage (Figure 7). In bag removal fruit, expression levels of anthocyanin biosynthesis genes are higher, whereas methyltransferase gene levels are lower than the levels in non-bagging fruit. In bagging fruit, most genes showed the opposite expression trends because there is no anthocyanin accumulation due to the lack of light exposure. The notable change in skin pattern from striped to blushed fruit is seen in bag removal fruit due to the re-exposure to light. Statistically significant differences in expression are also seen for anthocyanin-related genes between bag removal and non-bagging fruit. The regulatory genes *MdMYB10*, *MdWD*, and *MdGST* are significantly upregulated in bag removal fruit. Although the structural genes *MdLDOX* and *MdUFGT* are slightly downregulated in bag removal fruit, other structural genes are upregulated, especially *MdCHS*, *MdF3′H*, and *MdDFR*. Moreover, all methyltransferases are downregulated in bag removal fruit compared with non-bagging fruit. Statistically significant differences are seen for *MdCMT* and *MdDRM* genes. These results suggest that bag removal treatment induces the expression of anthocyanin biosynthesis genes and represses the expression of methyltransferases, resulting in red-blushed pigmentation on the fruit skin.

## 3. Discussion

### 3.1. Bag Treatment Induces Changes in Skin Color and Pattern

Sunlight is necessary to stimulate anthocyanin biosynthesis and accumulation in the skin of some red apple cultivars [26]. One approach for regulating fruit exposure to light in orchards is fruit bagging [11]. For example, bagging of ‘Geneva’ and ‘Pink Pearl’ apples decreased the anthocyanin accumulation to approximately 70% of that observed in unbagged fruit of the same cultivars [27]. Studies of bag treatment in non-red cultivars such as ‘Mutsu’, ‘Granny Smith’, and ‘Golden Delicious’ have shown that the fruit skin turned red or pink, but similar studies are lacking for striped apple cultivars [18,21,22]. In this study, we investigated the effects of bag treatment on anthocyanin biosynthesis regulation in apple skin and observed differences among the three treatment groups. Bag treatment of ‘Fuji’ apples caused skin color and pattern to change due to different accumulations of anthocyanin content. When bags were removed, the apples were re-exposed to sunlight, red pigmentation was stimulated, and the skin changed from a red-striped to a red-blushed pattern, suggesting the formation of striped and blushed patterns occur when anthocyanin is present in the peel [28]. However, light-induced anthocyanin accumulation was more prominent in bag removal fruit, causing the red-blushed skin pattern. In the bagging group, no anthocyanin was synthesized, resulting in yellow-blushed fruit. These results suggest that if there is no light, there is no red pigmentation [22]. Skin color parameters L*, a*, and b* were measured in non-bagging (red-striped), bag removal (red-blushed), and bagging (yellow-blushed) fruit samples by replacing the typical colorimetric method with the Tomato Analyzer software because the change in skin pattern requires an evaluation of the entire fruit surface [29]. The a*/b* ratio representing the redness was the highest in bag removal fruit, whereas the L* value representing the lightness was higher in bagging fruit. The increased value of a*/b* corresponding to the red-blushed pigmentation in bag removal fruit is consistent with the trend reported in a previous study that used color analyzer software developed in Matlab, which enabled the percentage of red to be calculated for ‘Honeycrisp’ apples having striped and blushed traits [30]. These findings also suggest that color measurement software with digital fruit images is efficacious for striped apple cultivars.

### 3.2. Bag Removal Promotes Anthocyanin Biosynthesis and Accumulation

Plants contain a variety of anthocyanin compounds [31,32]. The anthocyanin composition in apples is simple compared to other fruits [33]. Previous studies have shown that cyanidin-3-galactoside (cy-3-gal) is the principal pigment, accounting for more than 80% of the total anthocyanin composition [28]. Although the anthocyanin composition in the skin is similar among different apple cultivars, the skin appearance can differ. The same anthocyanin compounds are present in various skin color patterns. Different levels of cy-3-gal explain skin pigmentation variation [34]. Thus, our anthocyanin results were calculated as cy-3-gal. Anthocyanin content is the highest in bag removal fruit. An intermediate amount is seen in non-bagging fruit, and no anthocyanin accumulates in bagging fruit. These results suggest that red-blushed fruit has more anthocyanin and that the red-stripe pattern indicates regions where anthocyanin accumulation occurs. Most anthocyanin biosynthesis genes were upregulated in the non-bagging and bag removal samples, which was expected because they are red-colored. The level of anthocyanin steadily increased from S1 to S3 in non-bagging and bag removal samples, whereas the bagging group showed the opposite pattern. Compared to non-bagging fruit, the anthocyanin content of bag removal fruit was significantly higher, and that of bagging fruit was significantly lower, with the most significant differences at S3.

Our findings suggest that anthocyanin structural and regulatory genes exhibit distinct expression patterns in bag removal fruit. The differences in anthocyanin content are the most evident at S3 for each treatment group. A similar trend in anthocyanin accumulation was seen in anthocyanin-related genes. Regulatory genes *MdMYB10*, *MdWD*, and *MdGST* and structural genes *MdCHS*, *MdF3′H*, and *MdDFR* were significantly upregulated at S3. Early studies found that the structural genes encoding anthocyanin biosynthetic-related enzymes were upregulated in parallel with red pigmentation in apples unlike in pear and grape, where only the *MdUFGT* gene was the key structural gene to generate difference in anthocyanin accumulation [31,32]. Our results showed overall structural genes in the bag removal group were significantly upregulated during the fruit coloring period, especially in *MdCHS*, *MdF3′H*, and *MdDFR*. They were associated with significantly upregulated expression of *MdMYB10*, *MdWD*, and *MdGST*. When the fruits of ‘Mutsu’ apple were exposed to sunlight, the green/yellow skin turned red with light-induced expressions of nearly all anthocyanin biosynthesis genes including *MdCHS*, *MdF3H*, and *MdDFR* [22]. It is well known that the activity of structural genes was regulated by the transcription factor, primarily *MYB*, *bHLH*, and *WD* consisting of the MYB-bHLH-WD complex [35]. *MdMYB10* is a key gene in our study because we detected a rapid increase in expression that peaked at S3 in blushed apples than striped apples. *MYB*s have been identified as critical regulators in various fruit crops because the MBW complex coordinates the induction of anthocyanin biosynthesis [36]. These results suggested that the regulation of anthocyanin biosynthesis-related gene expression affects the amount of pigmentation in the apple skin, perhaps through *MdMYB10* regulatory genes.

### 3.3. Demethylation in the M7 Region of MdMYB10 Promoter Causes the Blushed Pattern in Bag Removal Fruit

DNA methylation, which is one of an epigenetic modification, has been suggested that was involved in the fruit color of woody plants, but the mechanism that mediates DNA methylation is not fully understood [37]. In this study, the ‘Fuji’ cultivar retaining red-striped skin on its fruit turned red-blushed skin with the upregulation of *MdMYB10* expression when the paper-bag was removed. The expression level of the *MdMYB10* transcription factor can be regulated by DNA methylation in the promoter region. Our results first obtained for *MdMYB10* promoter regions (M1-M7) showed that M2 (−856 to −383) and M7 (−2585 to −2117) regions were highly methylated regardless of treatment. Between parent and somatic mutant, the methylation level of *MdMYB1*/*10* genes has been examined. Contrary to our results, other regions −1246 to −780 have methylation enrichment in the ‘Blondee’ apple, which was yellow skin somatic mutant of ‘Kidd’s D-8’ [20]. That region also showed variable differences in methylation levels among the three cultivars: red-striped ‘Nagafu 2’ and ‘Yanfu 3’ and fully-red ‘Yanfu 8’ [24]. In ‘Honeycrisp’, another striped cultivar, a 900 bp region upstream of the translation start site is associated with increased methylation [19]. Similar to our results, the methylation of *MdMYB10* promoter has been observed in −856 to −383, −1246 to −780, and −2585 to −2117 regions in red-striped ‘Fuji’ apple [23]. The results suggested that the methylation in certain regions of the *MdMYB1*/*10* promoter caused anthocyanin deficiency on fruit skin during the coloring period. The notable region is M7 because this pattern of methylation in that region appears to be rare in other cultivars, unique in striped fruit of ‘Fuji’ [23]. Although almost complete digestion of DNA was detected at immature stages, the recovery of methylation was seen at the mature stage for successful amplification after the bag was removed. This demethylation level is caused by light stimulation, which is one of the environmental factors equal to the red-blushed fruit of ‘Beni Shogun’, bud mutant of ‘Fuji’ [23]. When the bag was removed, hypomethylation is detected especially in the CHG pattern. Although there is a dramatically lower CHH cytosine methylation in other red fruits [20,24], the importance of the CHG cytosine methylation in fruit coloration has been emphasized in ‘Fuji’ apples [23,38]. Since methylation in the M7 region is located upstream than the M2 region, the recovery of methylation would be correlated positively with *MdMYB10* expression and anthocyanin accumulation.

The mechanism of how promoter methylation is regulated did not fully elucidate, so additional study is needed. One of the reasons is the activities of DNA methyltransferases notably examined in Arabidopsis [17]. In apple, the family of methyltransferases and demethylase genes was first reported by transcriptome analysis [18]. Briefly, after treatment of 5-aza-2′-deoxycytidine acting as a DNA methylation inhibitor, expression levels of methyltransferases (*MET* and *DRM*) were downregulated while activities of demethylase genes (*DME* and *ROS*) were slightly induced in ‘Granny Smith’ cultivar. Although demethylase gene did not correlate with methylation levels in our result (Figure S2), the decreased expression level of methyltransferases (*MdMET*, *MdCMT*, and *MdDRM*) was detected in bag removal fruit. Unlike mammals, plants have a unique family of cytosine methyltransferase, *CMT* [17]. The expression level of *MdCMT* was first examined in this paper and also downregulated when the bag was removed. At the mature stage, the combination of lower expressions of *MdMET*, *MdCMT*, and *MdDRM* resulted in the methylation recovery of the *MdMYB10* promoter. These results may explain the production of the red-blushed pigmentation in bag removal fruit.

## 4. Materials and Methods

### 4.1. Plant Materials and Paper-Bagging Protocols

‘Fuji’, a commercial apple cultivar, was used in this study because its skin displays red-striped pigmentation. Apples were cultivated in the same orchard at the Apple Utilization Research Institute of Gyeongsangnam-do Agricultural Research & Extension Services in Geochang, Korea (35°43′ N, 127°54′ E, elevation 257 m) to minimize variations in the environmental conditions. Some apples were wrapped with double-layer paper bags (red translucent inner paper, white and black light-blocking outer paper) to regulate light exposure. Paper-bagging commenced on 1 August, 2020 before the beginning of fruit coloration and terminated on 5 October, 2020 (approximately 1 month before commercial harvesting), when the rate of anthocyanin accumulation was rapid. Bags were removed at intervals of one week (the outer bag on 28 September, 2020, the inner bag on 5 October, 2020) to prevent sunburn damage. Unbagged fruit grown under natural sunlight was used as the control group (Figure 1A, Non-bagging). Paper-bagged apples were either unbagged (Figure 1B, Bag removal) or not (Figure 1C, Bagging). In other words, apples in the bag removal group were re-exposed to natural sunlight, whereas apples in the bagging group were shielded from exposure to sunlight. Samples were harvested at three time points: 97 (S1), 169 (S2), and 191 (S3) days after full bloom (DAFB). S2 and S3 correspond to 1 and 4 weeks after bag removal. For each time point, 6 apples were collected from 4 trees in different locations (24 apples). Skins were peeled in a dark room to avoid light-induced responses during sampling, immediately frozen in liquid nitrogen, and stored at −80 °C. Skins were finely ground before analysis using a 6870 Freezer/Mill (SPEX SamplePrep, Metuchen, NJ, USA) with continuous addition of liquid nitrogen to maintain the temperature at −80 °C.

### 4.2. Measurement of Anthocyanin Content and Skin Color Parameters

Apple skin pigmentation was analyzed using two methods because bag treatment induces skin color and pattern changes.

Total anthocyanin was extracted by the pH-differential method using KCl buffer (0.025 M, pH 1.0) and NaAC buffer (0.4 M, pH 4.5) [39]. A structural transformation of anthocyanin occurs in response to a change in pH (colored at pH 1.0 and colorless at pH 4.5). Total anthocyanin was extracted from 0.5 g finely ground fruit peels in 5 mL of 1% HCl in methanol (*v*/*v*) for 24 h at 4 °C in darkness. Extracted anthocyanin was processed through a 0.5 μM syringe filter before anthocyanin analysis. The first 1 mL aliquot of fruit peel extract was transferred to a 15 mL tube, and 4 mL of KCl buffer was added. The second 1 mL aliquot of the fruit peel extract was placed in a 15 mL tube, and 4 mL of NaAc buffer was added. The solutions were mixed and extracted for 15 min at 4 °C in darkness. The absorbance of each solution was measured using a SmartSpec Plus spectrophotometer (Bio-Rad, Hercules, CA, USA) at 530 and 700 nm. The anthocyanin content formula was as follows: The monomeric anthocyanin pigment (mg/L) = (A × MW × DF × 103)/(ε × 1), where A is (A530 − A700) at pH 1.0 − (A530 − A700) at pH 4.5; MW is the molecular weight; DF is the dilution factor; ε is the molar absorptivity, and 1 is wavelength in cm. The anthocyanin content of three replicates was expressed as cyanidin-3-galactoside equivalent in mg/L because cyanidin-3-galactoside is the predominant anthocyanin in apples.

The color parameters L*, a*, and b* from an entire fruit skin surface image were estimated using Tomato analyzer 2.2 software [29]. To obtain values of L*, a*, and b*, images from a minimum of 24 fruit per sampling point were captured and estimated. For optimum color measurement, the illuminating system was calibrated to D65. Measurements from each fruit were entered into an Excel file and used to calculate L* and a*/b* values. The L* value indicates lightness in fruit skins, and the a*/b* ratio indicates redness [40].

### 4.3. RNA Extraction and Quantitative Real-Time PCR Analysis

Nucleic acids were extracted from ground fruit skins. In brief, total RNA was prepared using the CTAB-LiCl method [41], and cDNA was synthesized with a QuantiTect Reverse Transcription Kit (Qiagen, Germany) encompassing two reactions: first, the elimination of DNA, and, second, the reverse transcription of RNA. The concentration (µg/mL) and quality were determined by A260/A230 and A260/A280 ratios using a SmartSpec Plus spectrophotometer (Bio-Rad, Hercules, CA, USA) and visual verification on a 0.8% agarose gel.

Quantitative real-time PCR was performed using cDNA as a template with 2X qPCRBIO SyGreen Blue Mix Lo-ROX (BioD, Korea). The primers used are shown in Table S1. Three technical replicates for each reaction were analyzed with a Rotor-Gene Q (QIAGEN, Germany) and started with a preliminary step of 95 °C for 2 min followed by 35 thermal cycles of 95 °C for 10 s, 60 °C for 15 s, and 72 °C for 20 s. The gene transcript levels were measured, and relative quantification was determined with the 2^−^^△△^Ct algorithm.

### 4.4. DNA Extraction and Methylation Analyses

Genomic DNA (gDNA) was extracted using a DNeasy Plant Mini Kit (Qiagen, Germany), according to the manufacturer’s instructions after modifying for DNA extraction from apple fruit. gDNA was used for DNA methylation analyses after determining the concentration (µg/mL) and quality (A260/A230 and A260/A280 ratios) with a NanoDrop 2000 Spectrophotometer (Thermo Fisher Scientific, Waltham, MA, USA).

The McrBC-PCR method was used to analyze methylation levels of targeted gene promoter regions. In this analysis, 250 ng gDNA was digested with the methylation-specific endonuclease McrBC (New England Biolabs, Ipswich, MA, USA). For complete digestion, the reaction was incubated overnight at 37 °C and inactivated for 20 min at 65 °C. In a parallel negative control reaction, sterile distilled water was substituted for guanosine-5′-triphosphate (GTP). PCR was conducted with digested DNA from seven fragments (M1-M7) of the MdMYB10 promoter (M1, −440 to +1; M2, −856 to −383; M3, −1246 to −780; M4, −1657 to −1184; M5, −2044 to −1590; M6, −2255 to −1872; M7, −2585 to −2117) using previously published primers at 94 °C for 1 min, followed by 35 cycles at 94 °C for 30 s, 62 °C for 30 s, and 72 °C for 1 min, and finally at 72 °C for 10 min. The primers used for McrBC-PCR are shown in Table S1. The number of amplicons was visualized by agarose gel (1.5%) and used to evaluate the methylation status of their corresponding regions.

Mature apples were used for bisulfite sequencing to compare methylation levels among different bag treatment samples. Apple peel gDNA was used after ethanol precipitation for bisulfite sequencing. In addition, 500 ng gDNA was processed using an EpiTect Bisulfite Kit (Qiagen, Germany), according to the manufacturer’s instructions. The M7 region was amplified to detect differences in methylation level in detail. PCR was conducted using PCRBIO Ultra Polymerase (BioD, Korea) in a 50 µL reaction mixture (20 ng of treated DNA, 400 nM primer, 0.25 U of Taq polymerase). PCR included an initial denaturation at 95 °C for 1 min, followed by 35 cycles at 95 °C for 30 s, 56 °C for 30 s, and 72 °C for 1 min, and a final elongation step at 72 °C for 10 min. PCR products were purified using a Gel and PCR Purification System (BIOFACT, Korea) and cloned using a T-Blunt PCR Cloning Kit (SolGent, Korea). Ten clones were sequenced per sample and analyzed with the online software CyMATE [42].

### 4.5. Heat Map Analysis

A heat map of expression levels for anthocyanin- and methylation-related genes at the mature stage (S3) was constructed with the Heatmapper software [43]. The log_2_-transformed expression level is indicated using a color scale from −1 (green), through 0 (black), to +1 (red), representing low, intermediate, and high values, respectively.

### 4.6. Statistical Analysis

Significant differences among samples were assessed with the Student’s *t*-test (significance levels of *p* < 0.05 and 0.01 are indicated as * and **, respectively). Statistical analyses were performed using Microsoft Excel 2013.

## 5. Conclusions

In this study, paper-bagging followed by bag removal caused skin pigmentation to change from red-striped to red-blushed in ‘Fuji’ apples. During the fruit coloring period, nearly all regulatory and structural genes involved in the anthocyanin biosynthetic pathway were significantly upregulated after bag removal. The increased expression of the *MdMYB10* transcription factor was closely correlated with the red-blushed pattern in bag removal fruit. Our findings suggest that the re-exposure to sunlight induced methylation recovery in the M7 promoter region (−2585 to −2117). Furthermore, methyltransferase genes such as *MdMET*, *MdCMT*, and *MdDRM* might be related to decreasing methylation levels by downregulating gene expression. These data improve our understanding of the regulatory mechanisms of bag removal-induced skin patterning in ‘Fuji’ and possibly other striped apple cultivars.

## Data Availability

Not applicable.

## Supplementary Materials

The following supporting information can be downloaded at: https://www.mdpi.com/article/10.3390/ijms23063319/s1.
