# Supplementary material for: Effect of Paper-Bagging on Apple Skin Patterning Associated with MdMYB10 Promoter Methylation
Source: Int J Mol Sci. 2022 Mar 19;23(6):3319. doi: 10.3390/ijms23063319 (PMC8948882; doi:10.3390/ijms23063319)
Supplement: Supplementary file 1 [file ijms-23-03319-s001.zip › ijms-1637583-supplementary.pdf]

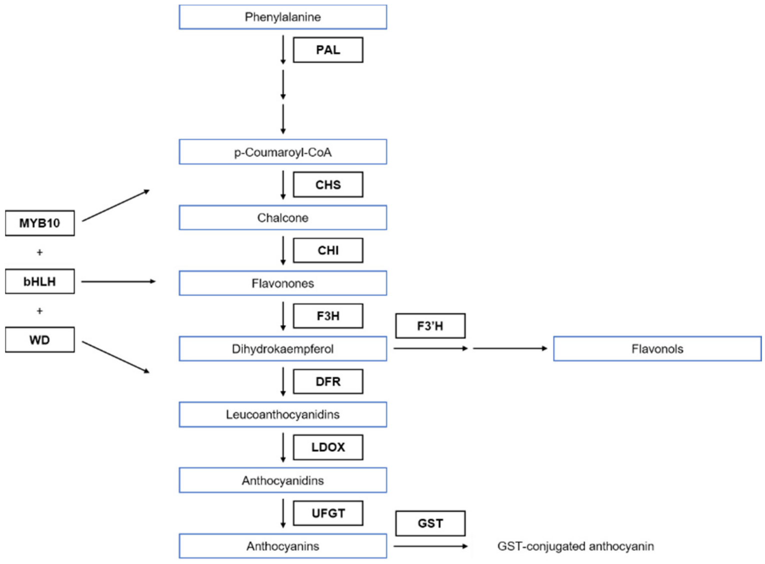

Figure S1. Diagram of the anthocyanin pathway in apple (*MYB10*, *bHLH*, and *WD* are transcription factor; *PAL*, phenylalani ne ammonia lyase; *CHS*, chalcone synthase; *CHI*, chalcone isomerase; *F3H*, flavone 3-hydroxylase; *F3'H*, flavonoid 3'-hydrox ylase; *DFR*, dihydroflavonol reductase; *LDOX*, Leucoanthocyanidin dioxygenase; *UFGT*, UDP-glucose: flavonoid 3-O-glucosyl transferase; *GST*, glutathione S-transferase).

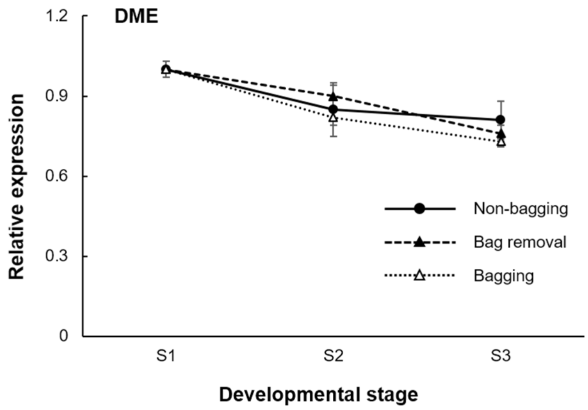

Figure S2. Quantitative real-time PCR analysis of demethylation-related gene, DEMETER (*DME*), in 'Fuji' apples cultivated under different bagging treatments: no bagging (control; labeled as "non-bagging"), bagging followed by bag removal (label ed as "bag removal"), and bagging with no bag removal (labeled as "bagging"). Apples were harvested at three time point s: before paper-bagging (S1, DAFB 97) and then 1 and 4 weeks after bag removal (S2, DAFB 169 and S3, DAFB 191). The x -axis and y-axis indicate the time of harvesting and relative expression, respectively. The expression level at S1 was set as 1 . Bars represent the standard error of triplicate samples. * and ** indicate significance levels of $p < 0.05$ and $p < 0.01$, respe ctively. DAFB, days after full bloom.

Table S1. Primers for quantitative real-time PCR and methylation analyses used in this study.

Gene name	Forward	Reverse
**Related to anthocyanin**		
Actin	CTGAACCCAAAGGCTAATCG	ACTGGCGTAGAGGGAAAGAA
MYB10	AGGCAGTGCGTTGAGATTC	CAGCTCTTCCTGCACCTGT
bHLH	AGAGGAGGAAGTTACCGGCT	CATGGCTCTGACCTGCATCT
WD	GCCATTTCATGGTCTCCAG	TCCAGGTCTCACCACATCC
PAL	AGGAACACCGTAAAGAACA	ACATACTCCCTATCGACAAC
CHS	AAGGACTCAAAACGACCGGG	TAAACCCACGCTGTGAAGCA
CHI	GTCAAGGAATTACCGACATTGA	CACAACTTCAGGGTCCAAGTAA
F3H	CTGGGGTATTTTCCAGATTGTT	TGGCAAAGCAAAGAACTCTCTA
F3′H	CAACAAGAGCTGGACCAAGTAG	TTACTGCTTGGAGGTAGGTCAA
DFR	GAGCAAGCTGCATGGAAAT	CATGAGAAATGGCCCAATC
LDOX	GTTCCAAATTCCATCGTCAT	TCACCTTTTCCTTGTTCACC
UFGT	TGCAGACCAGAGGCTTAATG	CCTCCCTGGTAAAAACTCCA
GST	GGAAGGCCACAACTTCAACG	TGGCTCAGATCAGCCAGAGT
**Related to methylation**		
Actin	CTGAACCCAAAGGCTAATCG	ACTGGCGTAGAGGGAAAGAA
MET	CGGAATAAGGGTGCTGAC	AGATACTCCTGGGAACGA
CMT	GTGCACTGTACACACCTCGT	ACTGCGTCGTAAACAGCGTA
DRM	GAGCAAACCAACCAGAG	ATCAAATCCGCCAAACT
DME	AGAATCCTGGGCTTGCTT	TCTCCATGCCTACGCTTT
**McrBC-PCR**		
M1 (−440 to +1)	GGTATCTTATGGTGGTCAAAGATG	CTTATCTGCTAGCAGCTAAGCTTA
M2 (−856 to −383)	CTGAGATTGACTCTTGTGAAAGCT	GTGAATGCAGAATCGTGTAACTAGT
M3 (−1246 to −780)	TTAACGGAATCCAACGAAGACAAG	GCTACACCTAACACATTGCTCAAT
M4 (−1657 to −1184)	TGAGATAGGTCCGGTTCTATTTCT	ATCCCTTTCCCTTATTTGTTCCGT
M5 (−2044 to −1590)	CAATTGCAGTGCTCAGAAATCGTT	CCGGACCCGTTTATAAATAGAACA
M6 (−2255 to −1872)	CCATTTCCACCGTTCATTTCTAAG	AACAGCAAACACCCAAAATCCCTT
M7 (−2585 to −2117)	CACTAGCTTCGGATTCCTTAGGA	CGGTTTAGTTTCTGGGAATTCACA
**Bisulfite sequencing**		
M7B	YGGATTTTTTAGGAATTTTGAAGTTAAG	AACAACAAACACCCAAAATCCCTTTAA
